# Oral Health Care of People with Down Syndrome in Germany

**DOI:** 10.3390/ijerph191912435

**Published:** 2022-09-29

**Authors:** Peter Schmidt, Laura C. Suchy, Andreas G. Schulte

**Affiliations:** 1 Department of Special Care Dentistry, Witten/Herdecke University, 58455 Witten, Germany; 2 Department of Child and Adolescent Psychiatry, Psychotherapy and Child Neurology, Gemeinschaftskrankenhaus Herdecke, 58313 Herdecke, Germany; 3 Department of Child and Adolescent Psychiatry, Witten/Herdecke University, 58455 Witten, Germany

**Keywords:** special care dentistry, tooth brushing behaviour, assisted oral care, caries prevention, fluoridated table salt, dental health care

## Abstract

Background: Due to a dearth of information on preventive and supportive oral care for people with Down Syndrome (PDS) in Germany, caregivers of PDS were surveyed on the topic. Methods: An anonymized questionnaire was sent to the 610 members of the German Down Syndrome Association. The study was approved by the ethics committee of Witten/Herdecke University (# 165/2017). Results: The parents/caregivers of 207 PDS responded. These PDS were between 1 and 55 years old (mean age 24.4 years) and included 111 males and 96 females. At their first dental appointment, 40.7% (*n* = 82) had been younger than three years. Although 78.3% (*n* = 162) tooth brushed twice daily, only 30.9% (*n* = 64) brushed for 2–3 min; 84.0% (*n* = 135) did not use other dental hygiene products apart from toothbrushes. Age-specific differences were found: Although only 30.8% (*n* = 12) of PDS < 18 years (*n* = 39) independently performed dental self-care; this was 67.3% (*n* = 113) for PDS ≥ 18 years (*n* = 168). A statistically significant (*p* < 0.001; Chi-Square-Test) difference in toothbrush preferences emerged: While PDS < 18 years mainly used manual toothbrushes, PDS ≥ 18 years preferred electric toothbrushes. In contrast to 72.6% of PDS ≥ 18 years, only 51.3% of PDS < 18 years accepted most types of toothpaste. Conclusions: Age-dependent differences in tooth brushing behaviour became evident within the group of PDS in Germany. Hence, better age-specific, inter-professional dental prophylaxis concepts need to be developed and offered for all age groups of PDS. These concepts should include personalized check-up intervals and hands-on instruction in dental hygiene procedures by dental practitioners.

## 1. Introduction

In 1866, the English physician J. Langdon Down first described the classic characteristics of Down Syndrome [1]. Later, it became known that persons with Down Syndrome (DS) carry three copies of chromosome 21, instead of two; moreover, in a small subgroup of this population (approximately 5%) the syndrome is caused by other chromosomal aberrations, such as translocation, mosaicism, or partial trisomy [2,3]. Although the term “Down Syndrome” has, strictly speaking, now been replaced by the medical term “Trisomy 21”, both terms are still used synonymously. In Germany, there are around 50,000 persons with Down Syndrome. Trisomy 21 (Q90.9, ICD-10 code; LD40.0, ICD-11 code) is thus the most frequently encountered viable constitutional chromosomal abnormality [4]. Characteristically, Trisomy 21 may be associated with different degrees of syndrome-related mental disability or intellectual impairment [3].

In the context of oral health, the varying degree of intellectual disability within the population of persons with Down Syndrome (PDS) is a highly pertinent aspect, as vast differences in the ability to independently manage adequate oral and dental self-care may be encountered within this group of people. Various national and international studies have identified a compromised capacity for dental self-care as one of the reasons why people with intellectual and other syndrome-related disabilities are seen to have poorer oral health than the general population [5,6,7,8,9,10,11,12]. Numerous systematic reviews have already been conducted on the prevalence of dental disease in persons with Down Syndrome and dental health care in this population. These reviews have covered a wide gamut of topics from implant provision [13], caries prevalence and caries experience [12,14,15], periodontal diseases [16], orthodontic palatal plate therapy [17], bruxism prevalence [18], and misaligned teeth [19] to general oral health issues pertinent to this patient population. Strikingly, none of these, however, provide explicit information on the status of caregiver-assisted, or supervised, oral hygiene for persons with Down Syndrome at home, or that of supportive oral hygiene interventions through professional dental providers (e.g., advice, demonstration, and training of oral hygiene procedures). Finally, over the last 20 years, studies investigating these aspects of dental care have begun to emerge from various countries around the globe [20,21,22,23,24,25,26,27,28,29,30,31]. In part, these studies are based on surveys of families with a family member with Down Syndrome. Similar studies and similar data are so far, however, lacking for Germany.

The main objective of the present study was, therefore, to acquire information on tooth brushing behaviour, supportive oral care interventions, and use of caries-preventive fluoride products in people with Down Syndrome (PDS) in Germany. A secondary objective was to see if there were age- or sex-related differences (i.e., children and adolescents vs. adults; male vs. female) in this regard.

## 2. Materials and Methods

The present study was conducted in collaboration with the *Arbeitskreis Down-Syndrom Deutschland e.V.*, a German Down Syndrome association. The members of this nationwide, non-profit association are primarily persons having a family member with PDS. Both the members and board of the association were willing to actively participate in the study.

***Questionnaire:*** The data were collected with a paper-and-pencil questionnaire that had been specifically designed by the study group. In the preparatory phase, questionnaire parameters such as type, execution, scope, and aim of the survey were defined in cooperation with the relevant contact persons from the association. In addition, the association’s members were informed that this study was in preparation and that they could indicate relevant items, which should be included in the questionnaire. A postal questionnaire comprising 75 questions (70 closed and 5 open) on the provision of dental health care was then complied for use in the study. From all questions, 12 of them related to sociodemographic aspects and 30 questions investigated the following aspects in regard to PDS:

- tooth brushing behaviour (6 questions),
- assisted oral health care at home and supportive oral hygiene interventions by
- dental practitioners (5 questions),
- use of fluoride, at home (3 questions) and
- use of dental services (dentist) (16 questions).

The present study is essentially based on the evaluation of these questions and each of these questions was answered in at least 95% of the returned questionnaires. Further information, e.g., on the other questions can be found in the thesis published by Suchy (2021) [32].

***Survey period and ethical aspects:*** The 610 members of the German Down Syndrome Association received a questionnaire, which they were asked to return after anonymous completion. The questionnaires were dispatched in November 2017. For organizational and data security reasons, the prepared envelopes were sent to the administration of the *Arbeitskreis Down-Syndrom Deutschland e.V.*, where the envelopes were then addressed and distributed to the members. All of the questionnaires were successfully dispatched. In February 2018, a written reminder was sent and the deadline for the return of the completed questionnaires was set for the middle of April 2018. This written reminder was also mailed by post to all of the association’s members. The study was approved by the ethics committee of Witten/Herdecke University (# 165/2017).

***Transfer of Data, and Statistics:*** The data were then digitized and processed using Excel 2016 (Microsoft Corp., Redmond, Washington, DC, USA) and statistically exploratory analysed using SPSS Version 25 (IBM Corporation, New York, NY, USA). Two persons of the study team did data entry and data verification manually independently. The first person digitized the data into an Excel file and checked it several times during the data entry process. Subsequently, a second person checked the entered data in the Excel file independently of the first person. To make this possible, all questionnaires were given an internal number, which was also transferred into the Excel file. The arithmetic mean, median (age), minimum, maximum, frequency distributions (standard deviation), and significance were determined. Subgroups were formed to allow comparison of differences, which were analysed exploratorily using the Chi-square test (*p* ≤ 0.01). To this end, the datasets were stratified according to age (<18 years old/≥18 years old) or sex (male/female).

It should be noted that, in the following, the persons with Down Syndrome are also referred to as study population. In contrast to this, persons who completed a questionnaire in regard to a family member with Down Syndrome are referred to as parents or caregivers representing the study participants. In Germany, parents customarily have custody of their children up to the age of 18. Adults who are incapable of making relevant decisions in regard to their own health, or finances, or similar—for example, due to intellectual disability—are given a court-appointed legal guardian, or legal representative. Frequently, in such case, this function is transferred to the parents when their children reach the age of majority, and the parents then officially become the legal guardians for their child. In principle, other persons, e.g., siblings, other family members, or state-appointed professional guardians, can also be appointed for this task.

## 3. Results

In total, 207 questionnaires returned and completed by parents or caregivers of PDS could be included. The response rate was thus 33.9%. The questionnaires were almost exclusively completed (97.5%) by parents of PDS (mother or father) (Table 1). The PDS (study population) consisted of 111 males and 96 females aged between 1 year and 55 years. Their mean age was 24.4 years (SD ± 9.0) and the age distribution was normally distributed. In all, 39 of the questionnaires (18.8%) were completed by parents or caregivers whose family members with Down Syndrome were children or adolescents (0 to 17 years). In this age group, there were 17 females and 22 males. The other questionnaires provided information on adult PDS (female: *n* = 79 and male: *n* = 89).

Almost all of the PDS had lived in Germany since birth (*n* = 203; 98%), and most (*n* = 205; 99%) were German citizens. Furthermore, the majority of PDS (*n* = 146; 70.5%) lived with their parents or other family members, irrespective of their age and sex. Only 4 PDS were reported to live on their own or 8 in shared accommodations/with a partner, respectively. Further details for the study population can be found in Table 2.

***Tooth brushing behaviour and assisted oral health care at home:*** The given responses indicated that 25.1% (*n* = 52) of the PDS had begun to receive regular dental hygiene care at home before their first birthday. In another 18.2% of the families, regular dental hygiene care had begun when the PDS had been two years old or older (Table 3). Most parents reported that the PDS in their family could clean their teeth on their own, without assistance (60.4%). In 38.6% of the cases, the PDS consistently needed assistance in cleaning their teeth. Nearly every second PDS who needed this type of support was consistently assisted by the same caregiver. It, however, also became apparent that children and adolescents (76.9%; *n* = 30) were more frequently assisted by the same caregiver than were adult PDS (43.5%; *n* = 73). This difference was, however, not statistically significant (*p* = 0.813). Although male PDS (55.0%) were more frequently assisted by the same caregiver than females (43.8%), this difference was also not statistically significant (*p* = 0.234). In regard to the frequency with which the PDS brushed their teeth, 78.3% (*n* = 162) of the parents responded that the PDS performed this task twice daily. Nearly identical percentages were found in this regard for the subgroups (i.e., male/female and children/adults). The majority of PDS (56.9%; *n* = 118) were found to brush their teeth for a duration of less than 2 min. Slightly less than one-third of the PDS (30.9%; *n* = 64) were found to brush their teeth for a duration of 2–3 min.

**Figure 1:**
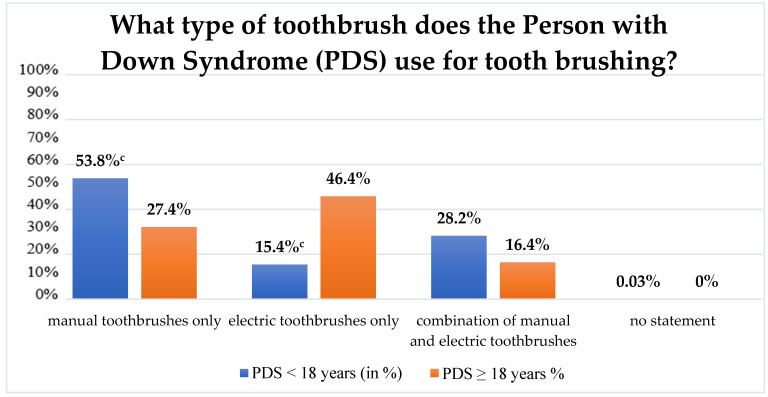
Percentage distribution of toothbrush types used by children and adolescents with Down Syndrome and by adults with Down Syndrome (^c^—*p* = 0.001).

**Figure 2:**
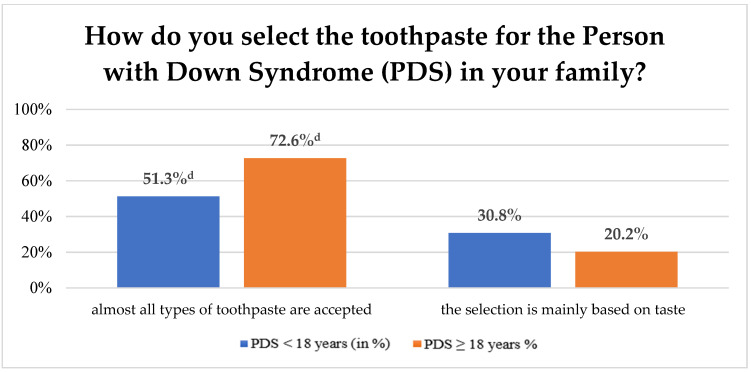
Percentage distribution of toothpaste preferences by children and adolescents with Down Syndrome and by adults with Down Syndrome (^d^—*p* = 0.101).

In response to which type of toothbrush the PDS used, a difference emerged between the age groups: While PDS younger than 18 years most frequently used manual toothbrushes (PDS < 18 years: 53.8%, *n* = 21; PDS ≥ 18 years: 27.4%, *n* = 46), PDS older than 18 years primarily used electric toothbrushes (PDS < 18 years: 15.4%, *n* = 6; PDS ≥ 18 years: 46.4%, *n* = 78) (Figure 1). This difference was statistically significant (*p* < 0.001). Apart from toothbrushes, only 4.7% (*n* = 9) of the PDS used dental floss to clean interdental spaces, and only 6.3% (*n* = 13) used interdental brushes for the same purpose. In contrast, 84.1% (*n* = 174) of the PDS did not use any other dental hygiene implements other than toothbrushes. This finding applied equally to both sexes and both age groups. Toothpaste types were primarily chosen on the basis of fluoride content or of flavour. A good two-thirds (68.6%; *n* = 142) of the PDS accepted almost all of the types of toothpaste on the German market. While this finding was true for almost three-quarters of the adult PDS, it applied for only every second PDS in the child/adolescent age group (Figure 2). Nonetheless, no significant difference was found in this respect between the two age groups (*p* = 0.101) or between the two sexes (*p* = 0.216). Further details on tooth brushing behaviour are given in Table 3.

***Use of fluoride in household products:*** In Germany, various products containing fluoride are available for domestic use that can help prevent caries. The questionnaire asked about three scenarios in this context (Table 3). For the entire group, two-thirds (65.2%; *n* = 135) of the responding parents/caregivers of persons with Down Syndrome affirmed that they used fluoridated table salt in the preparation of meals at home. The use of fluoride mouth rinses (20.3%; *n* = 42) or fluoride gels (12.6%; *n* = 26) was less common. Further details in this regard, for instance, for the two age groups, can be found in Table 3.

***The use of dental health services and professional supportive oral hygiene interventions:*** In the majority of cases (62.4%; *n* = 127), the PDS had reportedly been older than two years at their first dental appointment. Only 9.7% (*n* = 20) of the PDS under study had been to a dentist before the age of one year. Moreover, the responses showed that for the majority of the study population (all PDS: 87.9%, *n* = 182; PDS < 18 years: 89.7%, *n* = 35; PDS ≥ 18 years: 87.5%, *n* = 147), the primary reason for visits to the dentist were for check-up purposes. Another 8.3% named prophylaxis sessions as the reason for dental appointments. By and large, the responses were similar for both age groups (PDS < 18 years: 10.2%, *n* = 4; PDS ≥ 18 years: 8.3%, *n* = 14). Pain as a motive reason for the visit to the dentist was named only in the group of adult PDS (3.5%; *n* = 6).

Irrespective of age and sex the responses showed that the majority of the PDS (68.1%; *n* = 141) had been instructed in a dental practice, on how to brush their teeth. However, only 38.2% (*n* = 79) of the parents/caregivers reported that tooth brushing had been actively practised together with the PDS in a dental practice. Further details on supportive oral hygiene interventions by dental providers are presented in Table 4.

## 4. Discussion

The present study, which is based on national data from a postal questionnaire, for the first time permits assessment of the tooth brushing behaviour and preferences of persons with Down Syndrome in Germany, as well as their use of fluoride, as a caries preventive measure, at home. Additionally, this study provides information about home-based assisted oral health care through parents/caregivers and the use of dental health services and access to professional supportive oral hygiene interventions (e.g., advice, demonstration, and training of oral hygiene procedures), for persons with Down Syndrome. Over the last twenty years, comparable studies have already been published for France, Sweden, Turkey, Belgium, Ireland, Great Britain, Malaysia, Brazil, the USA, Kuwait, and Canada [20,21,22,23,24,25,26,27,28,29,30,31,33].

From our perspective, the response rate of 33.9% for the questionnaires in our study was gratifyingly high for German standards. Likewise, the response rate also represents a limitation, because it cannot be excluded that the group of people who responded differs socio-demographically from the group who did not respond. Consequently, the answers might have been somewhat different if, e.g., 90% of the members had returned the questionnaire. The response, or utilization, rates in international studies on oral health and parents’ perception of oral health in PDS range between 10% and 100% [22,24,25,26,27,28]. This range in response rates for the different studies is, in particular, due to different methodologies. In some of the studies referred to above, a specific sample group (e.g., families with children or adolescents with DS) or sample size were predetermined [21,26]. In these approaches, specific families were approached and chosen as study participants. This method of directly approaching possible candidates will likely have had a positive effect on the willingness of candidates to participate in the studies. A methodological approach such as this facilitates gathering of data also for a comparison or control group (e.g., siblings without Down Syndrome) [26,27,33]. In our study, however, this approach was precluded by organizational, data security, and financial reasons. Moreover, the aim of our study was to survey not only pre-specified sample groups, but families with PDS of all ages. It is interesting that most of the studies published so far have been based on data for children or adolescents with Down Syndrome [21,22,23,24,25,28,29,30,31,33]. Our study is hence one of the very few to present data also for adult PDS. In light of the significant rise in life expectancy for people with Down Syndrome over the last years and the concomitant changes in the medical and social needs of these persons, this is a relevant aspect [34,35].

It is our belief that our early collaboration with the German Down Syndrome Association in preparing the survey was instrumental in raising awareness for the study. For example, information on the background and methods of the study was presented in several editions of the Association’s magazine before the study was conducted. Moreover, in the spirit of participative research, in edition No. 98 (2/2017) of the German Down Syndrome Association’s magazine, the association’s members were invited to help design questions for the survey. The study participants thus had the opportunity to suggest themes that they considered relevant to the provision of dental care for PDS. It is also important to note, that the authors of this study received numerous messages from members of DS association indicating that the topic of dental care is, considered highly relevant by families with a family member with Down Syndrome. In addition, many participants also viewed the circumstance that a survey on this topic was being conducted in Germany for the first time very positively. This can also explain why all questionnaires were almost entirely completed.

An important insight gained from this study is that only two-thirds of the surveyed parents of PDS (66.6%; *n* = 138) had begun to brush their children’s teeth regularly before the child’s first birthday. Interestingly, this finding was independent of sex or the current age of the PDS. In the authors’ view, regular oral hygiene routines should be introduced early in a child’s life so that babies and toddlers can already get used to the sensation of implements such as toothbrushes in their mouths, with a minimum of stress. At the latest when a baby’s first primary teeth erupt tooth brushing ought to be a routine procedure. Through their clinical experience, the authors of this study are well aware that dental health care and dental hygiene are not topics of primary concern for young parents during the first months or years of their child’s life—particularly not if they have a child with a congenital disability. In light of the various other general health issues that may exist, topics such as dental health and dental appointments take a back seat. This perception is supported by the responses to our survey in regard to PDS’s age at first dental appointment: The majority of the PDS were two years old or older at their first visit to the dentist (Table 4). Some studies conducted on German toddlers without underlying diseases, however, demonstrate that timely visits to dentists, already during babyhood, can help prevent early childhood caries [36,37,38]. In Germany, there are specialized paediatric centres for developmental problems, for example, for children born with disabilities. These centres provide support also for the families of children with genetic disorders such as Down Syndrome [39]. Although the care in these centres is fundamentally provided by inter-professional teams (physicians, therapists, speech therapists, etc.), to date, dentists are not yet included in these teams. The authors have called attention to this omission in another place [40].

An important parameter of dental self-care is the frequency with which teeth are brushed. In this study, it emerged that, irrespective of their age, almost all of the PDS (78.3%) brushed their teeth twice daily. A similar percentage (85.1%) has been reported from Sweden [21]. With 43%, respectively, 55%, considerably lower percentages of children with Down Syndrome who brushed their teeth twice daily are reported in comparable studies from Belgium and Brazil [23,41]. Similarly low percentages were found in another study, not only for persons with Down Syndrome (58%), but also for persons with cerebral palsy (30%) [42]. It is encouraging to see that more recent studies report a rise in the percentage of PDS who brush their teeth twice daily. The recommendation to brush twice daily is given in a German guideline for caries prophylaxis in permanent teeth. In addition, this guideline recommends to use fluoridated toothpaste [43]. Another German guideline additionally recommends that teeth should be brushed for the duration of at least two minutes, irrespective of the type of toothbrush used [44]. Less than a third of the PDS in our study, however, were estimated to be capable of brushing their teeth for two to three minutes (Table 3). Possible reasons for this low percentage may be that the duration of brushing subjectively seems longer than it actually is or that the result of brushing is prematurely felt to be adequate. Clinical experience shows that, in many cases, people with intellectual disabilities may also lack the perseverance to brush their teeth for two minutes. Additionally, possible impairments in motor skills or in the ability to concentrate may affect the duration of brushing and brushing behaviour.

Preferences such as the choice of toothbrush and toothpaste type must also be addressed as factors that impact oral hygiene. More and more, it seems that electric toothbrushes are, after all, suitable for use by PDS. Electric toothbrushes are more forgiving of brushing-technique errors than are manual toothbrushes. Additionally, they have supportive features such as integrated automatic pressure controls and timers. Our study showed that although electric toothbrushes were used by PDS, the frequency of their use was not uniform across all age groups: While PDS younger than 18 years primarily used manual toothbrushes to clean their teeth, PDS older than 18 years chiefly preferred electric toothbrushes (Figure 1 and Table 3). This difference between the two age groups was statistically significant and concurs with other reported findings in the literature [23]. Currently, the evidence supporting the possible superiority of electric toothbrushes over manual toothbrushes in oral care is, however, still inconclusive. Although electric and manual toothbrushes are found to be similarly effective for reducing gingival inflammation in people with intellectual disability in the medium term [45], electric toothbrushes are, for example, considered superior because they are said to more effectively reduce gingivitis [46]. This latter aspect can be quite relevant for PDS due to their physiological syndrome-related significant risk of developing periodontitis [47,48]. Nonetheless, based on the clinical experiences of the author’s, electric toothbrushes cannot be generally recommended for all people with Down Syndrome. Family members of persons with intellectual disabilities, for example, report that this subgroup may experience the particular noises that electric toothbrushes make as acutely unpleasant and thus reject using them. A possible topic for future research could, hence, be to determine age-specific criteria for the choice of suitable toothbrushes for PDS.

Our study showed that almost 70% of the surveyed PDS essentially accepted almost all types of toothpaste. Interestingly, however, while this was true for almost three-quarters of the adult PDS, this applied for only every second child or adolescent with DS. As it is known that children and adolescents, also those without underlying disorders or disabilities, often experience the flavour of some toothpastes as to too sharp, it is not surprising that a higher percentage of children and adolescents with Down Syndrome than adults report that they choose toothpastes on the basis of taste (Figure 2). Only roughly one-quarter of the study participants said they chose their toothpaste on the basis of whether it contained fluoride or not. From a scientific point of interest, it is notable to find that fluoride content, therefore, still appears to be of only secondary interest in the choice of a toothpaste. In light of these results, we have decided to explicitly ask in future studies if the participants use fluoride toothpaste or not. A recent publication by Krause et al. (2022) reports that 90% of the children and adolescents, in almost all age groups (0.5 to 17 years old), in the general population in Germany typically use a fluoride toothpaste [49].

As water fluoridation has had no chance to be permitted in Germany, salt fluoridation was introduced in 1991 in this country [50]. Quite generally, here the use of fluoridated table salt has risen over the last years, and it is now widely used [50]. In the present study, almost two-thirds (65.2%) of the participants affirmed that they used fluoridated table salt at home. For the PDS in the childhood and adolescent age group, this percentage was even slightly higher yet (Table 3). Based on the data from the current German epidemiological oral health study (DMS V), our findings in this regard are in keeping with those for the general population in Germany [51].

According to their parents or caregivers, a third of the adult PDS (34.5%; *n* = 58) in our study, however, still needed assistance with cleaning their teeth. From clinical experience, we would have expected the number of adult PDS who required support with their oral hygiene to be considerably higher. This discrepancy may, however, be due to various possible “selection biases” (both in regard to the participants in the study and the clinical experiences of the authors). On the one hand, it is conceivable that among the participating parents/caregivers there were a high number of those whose family members with Down Syndrome had a certain level of self-sufficiency in respect to dental- and oral hygiene. On the other hand, it is possible that the authors mainly see PDS with a high level of need for dental treatment in the dental clinic and with great difficulties with dental and oral hygiene care. Additionally, the author’s team acknowledges that there might be a scattered proportion of respondents who gave more positive answers about the PDS’s self-reliance in cleaning their teeth (over-reporting—relative to the truth), as likely exists for any population.

In the study, for the age group of PDS younger than 18 years, slightly over half of the parents (56.4%) indicated that the PDS in their family needed to be assisted with their dental hygiene. A Swedish survey that was conducted on the parents of children or adolescents with Down Syndrome reports that 72.3% of the parents assisted their children in cleaning their teeth on a daily basis [21]. By comparison, 92.3% of children with cerebral paresis receive this kind of support [20,42]. Fundamentally, the age of the PDS appears to be significant in regard to whether or not he or she received support in cleaning their teeth. Although children without disability are frequently considered capable of brushing their teeth on their own around the age of eight/nine years old, when they have learned to write fluidly in cursive script, PDS may have syndrome-related intellectual impairment, as mentioned previously. In our study, all of the children (*n* = 6) capable of always independently brushing their teeth were older than 10 years of age. Other studies have found similar results: A Belgian study, thus, found that 64% of children with Down Syndrome older than 10 years of age were no longer assisted in brushing their teeth everyday [23]. A Swedish study also found that the proportion of parents who supported their child with this task diminished with increasing age of the children [21]. This finding is also in accord with the results from our study. Furthermore, the findings of a Turkish study show that the average age of PDS who do not receive any kind of support with their dental hygiene is higher than that of those who are actively supported with this task [22].

In respect to the use of dental services, almost all of the parents stated that the primary reason for visits to the dentist with their family member with Down Syndrome was for check-ups or for prophylaxis sessions. While this finding is consistent with data from other European countries [21,23,25,26], it is not consistent with data from non-European countries. Shyama et al. (2015) thus report that, in Kuwait, the primary reason for dentist visits by PDS is dental pain or the need for dental restorations [31]. A Brazilian study even reports that almost 11% of the PDS in their study collective had never been to a dentist [29]. Furthermore, in a survey from the United States, 60% of the parents of PDS reported that inadequate financial compensation was the most frequent reason why dentists refused to treat PDS [30]. The latter studies illustrate that preventive healthcare systems, also in regard to dental health, are not yet established everywhere, nor is affordable preventive dental care available to most of the population in every country. These observations suggest that the significance of dental medicine and oral health for overall health and well-being is considerably underestimated in many countries. In Germany, in contrast, because of the system of statutory health insurance, roughly 90% of the population have access to preventive dental healthcare services. In addition, around 30 years ago, the statutory health insurances began to cover the costs for various caries preventive measures for all children and adolescents. The interested reader can find this topic covered more expansively in other publications by the current authors [8,52].

Because, in many cases, caregivers need to actively assist with daily oral hygiene procedures and sometimes even need to perform this task for the PDS, it is important to stress that both PDS, in all age groups, and their caregivers ought to be professionally instructed in proper tooth brushing techniques by dental practitioners within the scope of supportive oral hygiene interventions. The finding that this had been the case in a solid two-thirds of the participating parents/caregivers of PDS is encouraging (Table 4). However, only slightly over a third of the respondents reported that proper tooth brushing had been actively practised with the PDS in the dental practice. This percentage was essentially the same for both sexes and both age groups (Table 4).

## 5. Conclusions

For all age groups, the tooth brushing habits and oral self-care behaviour of people with Down Syndrome (PDS) to some extent differ from those of people without disability. Furthermore, within the group of PDS, age-related differences in tooth brushing behaviour are found, such as preferences for a certain type of toothbrush or type of toothpaste. Fundamentally, no sex-related differences were found in this respect. Some PDS may, thus, remain incapable of independently carrying out oral hygiene procedures even as adults. In these cases, life-long homebased, as well as professional, oral health support, e.g., in cleaning their teeth, is essential. In consequence, the efforts to develop and offer age-specific and more inter-professional dental prophylaxis concepts for PDS in all age groups, from infancy to senescence, must be increased. These concepts ought to encompass personalised check-up intervals and hands-on education and training in oral hygiene through dental staff.

## Figures and Tables

**Table 1:** Characteristics of the people who completed the questionnaire (family member/caregiver of the PDS).

Characteristics of People Who Completed the Questionnaire (Study Participants)		
**Relationship to person with PDS**		
mother	174	84.1%
father	28	13.5%
other relationship	1	0.5%
no statement	4	1.9%
**Age (in years)**		
mean ± (SD) median all	56.9 ± 10.1 56.0	
range	33–87	
age mothers (in years)		
mean ± (SD) median	55.8 ± 9.9 55.0	
range	33–87	
age fathers (in years)		
mean ± (SD) median	64.2 ± 8.7 66.0	
range	43–81	
age other relationship (in years)	72.0	
age no statement	60.0	

**Table 2:** Characteristics of the People with Down Syndrome (PDS) as the study population.

People with Down Syndrome (PSD)	Frequencies	*n* = 207
**Sex**		
Male	111	53.6%
Female	96	46.4%
**Age (in years)**		
mean all ± (SD) median all range (*n* = 207)	24.4 ± 9.0 25.0 1–55	
mean all (male) ± (SD) median all (male) range (male, *n* = 111)	23.9 ± 8.8 25.0 1–52	
mean all (female) ± (SD) median all (female) range (female, *n* = 96)	25.1 ± 9.3 25.0 3–55	
mean all < 18 years ± (SD) median all (<18 years) range (*n* = 39)	11.1 ± 4.5 12.0 1–17	
mean male < 18 years ± (SD) median male (<18 years) range (male, *n* = 22)	11.3 ± 4.1 12.0 1–17	
mean female < 18 years ± (SD) median female (<18 years) range (female, *n* = 17)	10.9 ± 4.8 13.0 3–17	
mean all ≥ 18 years ± (SD) median all (≥18 years) range (*n* = 168)	27.5 ± 6.8 26.0 18–55	
mean male ≥ 18 years ± (SD) median male (≥18 years) range (male, *n* = 89)	27.0 ± 6.6 26.0 18–52	
mean female ≥ 18 years ± (SD) median female (≥18 years) range (female, *n* = 79)	28.1 ± 6.9 27.0 18–55	
**Legal guardian**		
with guardian	160	77.3%
without guardian	40	19.3%
no statement	7	3.4%
**Living situation**		
Alone	4	1.9%
at parents’ home/at a family member’s home	146	70.5%
in supervised living	42	20.3%
in shared accommodation or with a partner	8	3.8%
Other	5	2.5%
no statement	2	1.0%

**Table 3:** Tooth brushing preferences and fluoride use of the People with Down Syndrome (PDS), as reported by the person who completed the questionnaire.

	All PDS (*n* = 207)	Male (*n* = 111)	Female (*n* = 96)	<18 years (*n* = 39)	≥18 years (*n* = 168)
At what age did tooth brushing start for your family member with Down Syndrome?					
in the first year of life	25.1% (52)	22.5% (25)	28.1% (27)	15.3% (6)	27.4% (46)
at the age of 1 year	41.5% (86)	44.1% (49)	38.5% (37)	53.8% (21)	38.7% (65)
at the age of 2 years	14.4% (30)	13.5% (15)	15.6% (15)	15.4% (6)	14.3% (24)
at the age of 3 years	3.8% (8)	5.4% (6)	2.1% (2)	2.6% (1)	4.2% (7)
I do not remember	12.0% (25)	10.8% (12)	13.5% (13)	5.1% (2)	13.7% (23)
no statement	2.8% (6)	3.6% (4)	2.1% (2)	7.6% (3)	1.8% (3)
Does your family member with Down Syndrome receive assistance in tooth brushing? *					
brushes teeth alone	60.4% (125)	58.6% (65)	62.5% (60)	30.8% (12)	67.3% (113)
needs assistance with tooth brushing	38.6% (80)	41.4% (46)	35.4% (34)	56.4% (22)	34.5% (58)
receives assistance with tooth brushing	18.4% (38)	16.2% (18)	20.8% (20)	41.0% (16)	13.1% (22)
receives assistance with tooth brushing at least once a week	10.1% (21)	12.6% (14)	7.3% (7)	2.6% (1)	11.9% (20)
Who assists with tooth brushing?					
usually the same person	49.8% (103)	55.0% (61) ^b^	43.8% (42) ^b^	76.9% (30) ^a^	43.5% (73) ^a^
almost always several persons	8.7% (18)	7.2% (8)	10.4% (10)	7.7% (3)	8.9% (15)
does not apply	41.5% (86)	37.8% (42)	45.8% (44)	15.4% (6)	47.6% (80)
How often does your family member with Down Syndrome usually brush teeth?					
once a day	14.5% (30)	14.4% (16)	14.6% (14)	12.8% (5)	12.9% (25)
twice a day	78.3% (162)	78.4% (87)	78.1% (75)	79.5% (31)	78.0% (131)
three times a day	4.8% (10)	4.5% (5)	5.2% (5)	7.7% (3)	4.2% (7)
after every meal	1.0% (2)	0.9% (1)	1.0% (1)	-	1.2% (2)
teeth cannot be brushed regularly	1.0% (2)	1.8% (2)	-	-	1.2% (2)
no statement	0.5% (1)	-	1.0% (1)	-	0.6% (1)
Please estimate how long your family member usually accepts tooth brushing?					
less than 1 min	7.7% (16)	8.1% (9)	7.3% (7)	10.3% (4)	7.1% (12)
1–2 min	49.2% (102)	51.4% (57)	46.9% (45)	51.3% (20)	48.8% (82)
2–3 min	30.9% (64)	27.9% (31)	34.4% (33)	25.6% (10)	32.1% (54)
this is different every time	10.6% (22)	10.8% (12)	10.4% (10)	10.3% (4)	10.7% (18)
no statement	1.4% (3)	1.8% (2)	1.0% (1)	2.6% (1)	1.2% (2)
Are additional dental hygiene implements used apart from the tooth-brush? *					
Interdental brushes	6.3% (13)	5.4% (6)	7.3% (7)	12.8% (5)	4.8% (8)
dental floss	4.3% (9)	3.6% (4)	5.2% (5)	-	5.4% (9)
dental woods	-	-	-	-	-
no additional implements	84.0% (174)	82.9% (92)	85.4% (82)	84.6% (33)	83.9% (141)
other additional implements	2.8% (6)	5.4% (6)	-	2.6% (1)	3.0% (5)
no statement	2.8% (6)	2.7% (3)	3.1% (3)	-	3.6% (6)
Are any fluoride products used for caries prevention? *					
mouth rinse with fluoride	20.3% (42)	21.6% (24)	18.8% (18)	7.7% (3)	23.2% (39)
gels with fluoride	12.6% (26)	8.1% (9)	17.7% (17)	12.8% (5)	12.5% (21)
table salt with fluoride	65.2% (135)	65.8% (73)	64.6% (62)	71.8% (28)	63.7% (107)
no use of these fluorides	21.3% (44)	21.6% (24)	20.8% (20)	23.1% (9)	20.8% (35)
Did your family member receive fluoride tablets as a child?					
yes	65.7% (136)	65.8% (73)	65.6% (63)	61.5% (24)	66.7% (112)
no	25.6% (53)	25.2% (28)	26.0% (25)	28.2% (11)	25.0% (42)
I do not remember (anymore)	6.3% (13)	5.4% (6)	7.3% (7)	7.7% (3)	6.0% (10)
no statement	2.4% (5)	3.6% (4)	1.0% (1)	2.6% (1)	2.4% (4)

* multiple responses possible; ^a^—*p* = 0.813; ^b^—*p* = 0.234.

**Table 4:** Information about the professional dental care for the People with Down Syndrome (PDS), as reported by the person who completed the questionnaire.

	All PDS (*n* = 207)	Male (*n* = 111)	Female (*n* = 96)	<18 years (*n* = 39)	≥18 years (*n* = 168)
When was your family member’s first visit to the dentist?					
in the first year of life	9.7% (20)	13.5% (15)	5.2% (5)	10.2% (4)	9.5% (16)
at the age of 1 year	12.1% (25)	8.1% (9)	16.7% (16)	10.2% (4)	12.5% (21)
at the age of 2 years	18.9% (37)	16.2% (18)	19.9% (19)	33.3% (13)	14.3% (24)
at the age of 3–5 years	35.3% (73)	36.9% (41)	33.3% (32)	33.3% (13)	35.7% (60)
at the age of 6–10 years	8.2% (17)	9.1% (10)	7.3% (7)	7.7% (3)	8.3% (14)
I do not remember	14.9% (31)	15.3% (17)	14.6% (14)	-	18.4% (31)
no statement	1.9% (4)	0.9% (1)	3.1% (3)	5.1% (2)	1.2% (2)
Were tooth brushing techniques explained and demonstrated to the family member at the dental practice?					
yes	68.1% (141)	72.1% (80)	63.5% (61)	56.4% (22)	70.8% (119)
no	30.4% (63)	27.0% (30)	34.4% (33)	41.0% (16)	28.0% (47)
no statement	1.4% (3)	0.9% (1)	2.1% (2)	2.6% (1)	1.2% (2)
Was tooth brushing practised with your family member at the dental practice you visited?					
yes	38.2% (79)	40.5% (45)	35.4% (34)	35.9% (14)	38.7% (65)
no	60.9% (126)	58.6% (65)	63.5% (61)	64.1% (25)	60.1% (101)
no statement	1.0% (2)	0.9% (1)	1.0% (1)	-	1.2% (2)
Were tooth brushing techniques also explained and demonstrated to the caregiver who assists the PDS with dental care at the dental practice you visited?					
yes	42.0% (87)	44.1% (49)	39.6% (38)	48.7% (19)	40.5% (68)
no	31.9% (66)	33.3% (37)	30.2% (29)	38.5% (15)	30.4% (51)
does not apply, as no supportive dental care is provided	22.7% (47)	20.7% (23)	25.0% (24)	12.8% (5)	25.0% (42)
no statement	3.4% (7)	1.8% (2)	5.2% (5)	-	4.2% (7)

## Data Availability

Due to the strict European General Data Protection Regulation and the statement in the questionnaire to the study participants, no data will be passed on to third parties. The dataset generated from this study cannot be deposited in a public repository, because the study participant consent did not include data sharing permissions. A request for access to data for researchers who meet criteria for access to confidential data must be made to the first author: Peter Schmidt, email: peter.schmidt@uni-wh.de, or to a representative of our Department of Special Care Dentistry, Dental School, Faculty of Health, Witten/Herdecke University, Germany and the board of the German Down Syndrome Association, Bielefeld, Germany, too.
